# Optimizing β-TCP with E-rhBMP-2-infused fibrin for vertical bone regeneration in a mouse calvarium model

**DOI:** 10.1093/rb/rbae144

**Published:** 2025-02-11

**Authors:** Kun Zhao, Mitsuaki Ono, Xindi Mu, Ziyi Wang, Shichao Xie, Tomoko Yonezawa, Masahiro Okada, Takuya Matsumoto, Takuo Kuboki, Toshitaka Oohashi

**Affiliations:** Department of Molecular Biology and Biochemistry, Okayama University Graduate School of Medicine, Dentistry and Pharmaceutical Sciences, Okayama, 700-8558, Japan; Department of Oral Rehabilitation and Regenerative Medicine, Okayama University Graduate School of Medicine, Dentistry and Pharmaceutical Sciences, Okayama, 700-8558, Japan; Department of Molecular Biology and Biochemistry, Okayama University Graduate School of Medicine, Dentistry and Pharmaceutical Sciences, Okayama, 700-8558, Japan; Department of Molecular Biology and Biochemistry, Okayama University Graduate School of Medicine, Dentistry and Pharmaceutical Sciences, Okayama, 700-8558, Japan; Department of Biomaterials, Okayama University Graduate School of Medicine, Dentistry and Pharmaceutical Sciences, Okayama, 700-8558, Japan; Department of Molecular Biology and Biochemistry, Okayama University Graduate School of Medicine, Dentistry and Pharmaceutical Sciences, Okayama, 700-8558, Japan; Division of Dental Biomaterials, Tohoku University Graduate School of Dentistry, Miyagi, 980-8575, Japan; Department of Biomaterials, Okayama University Graduate School of Medicine, Dentistry and Pharmaceutical Sciences, Okayama, 700-8558, Japan; Department of Oral Rehabilitation and Regenerative Medicine, Okayama University Graduate School of Medicine, Dentistry and Pharmaceutical Sciences, Okayama, 700-8558, Japan; Department of Molecular Biology and Biochemistry, Okayama University Graduate School of Medicine, Dentistry and Pharmaceutical Sciences, Okayama, 700-8558, Japan

**Keywords:** BMP-2, fibrin, vertical bone regeneration, biocompatible materials

## Abstract

Effective reconstruction of large bone defects, particularly in thickness, remains one of the major challenges in orthopedic and dental fields. We previously produced an *Escherichia coli*-based industrial-scale GMP-grade recombinant human bone morphogenetic protein-2 (E-rhBMP-2) and showed that the combination of E-rhBMP-2 with beta-tricalcium phosphate (β-TCP/E-rhBMP-2) can effectively promote bone reconstruction. However, the limited mechanical strength and poor morphology retention of β-TCP granules are key points that need optimization to obtain more effective grafts and further expand its clinical applications. Therefore, we combined β-TCP/E-rhBMP-2 with fibrin gel to enhance its mechanical properties and usability for vertical bone regeneration. We investigated the mechanical properties and vertical bone regeneration effects of the materials applied, with or without fibrin containing E-rhBMP-2, in a calvarial defect model in mice. Compression tests were conducted to assess the initial stability of the materials. Scanning electron microscopy and Fourier transform infrared spectroscopy were conducted to characterize the presence of fibrin on the scaffold. After 4 and 12 weeks of implantation, micro-computed tomography and histological and immunofluorescent analyses were performed to assess the morphology and volume of the newly formed bone. The fibrin-containing groups had significantly higher initial mechanical strength and higher ability to maintain their morphology *in vivo* compared to the counterparts without fibrin. However, fibrin gel alone suppressed the bone formation ability of β-TCP/E-rhBMP-2 whereas the presence of high doses of E-rhBMP-2 in fibrin gel resulted in material resorption and enhanced new bone formation. In conclusion, fibrin gel significantly improved the mechanical strength and surgical manageability of the β-TCP/E-rhBMP-2 scaffold, and the addition of E-rhBMP-2 to the fibrin gel further enhanced the vertical bone regeneration and initial structural integrity of the scaffold.

## Graphical abstract

**Figure:**
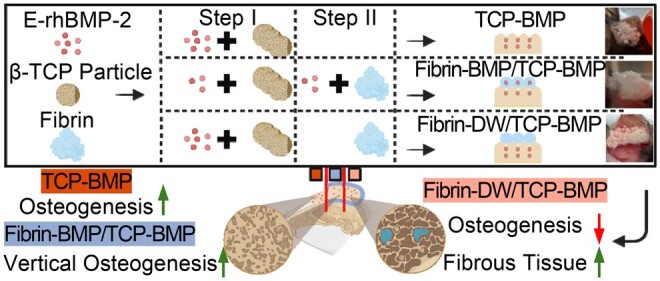

## Introduction

Bone reconstruction involves the utilization of various materials to promote the healing and restoration of bone tissue. Commonly used materials include autografts and allografts, as well as synthetic materials like hydroxyapatite (HA) and beta-tricalcium phosphate (β-TCP) [1]. However, autologous bone grafting requires additional surgery because the graft must be harvested from a donor site, which can lead to various complications [2]. In addition, allografts, xenografts and artificial bones, such as HA and β-TCP, have markedly lower osteoinductive capacity than autografts [3, 4]. Therefore, recent research has focused on the combination of different materials that load growth factors to promote bone regeneration, such as collagen-HA composites and silk fibroin/carbon nanofiber composites [5–8]. Such composite materials can provide structural support, promote osteogenic activity and improve bioactivities by successfully synergizing the advantages of various materials. For this reason, various research and clinical trials on composite materials are currently ongoing [9–11].

Previously, our research group successfully produced good manufacturing practices (GMP)-grade recombinant human bone morphogenetic protein-2 (E-rhBMP-2) using the *Escherichia coli* system for production at an industrial scale [12, 13]. Bone morphogenetic proteins (BMPs) are multifunctional growth factors belonging to the transforming growth factor-beta (TGF-β) superfamily and play an important role in bone remodeling [14]. Compared to BMP-2 derived from mammalian cells, E-rhBMP-2 performs similar functions but can be produced in larger quantities at a lower cost [15]. To improve its biofunctionality, we adsorbed E-rhBMP-2 onto β-TCP and after confirming its efficacy and safety in nonclinical studies [16], we started a clinical trial, in 2021, to reconstruct the maxillary and mandibular bone before the surgical placement of oral implants in Japan. However, although β-TCP granules have a compressive strength of about 5 megapascals (MPa), there is no binding between them. As a result, β-TCP granules cannot be freely molded as a block and do not retain the fixed morphology, leading to technical difficulties during material implantation. Furthermore, if the material is extruded beyond the implantation site, there is a risk of ectopic ossification [17].

Fibrin gel has been clinically applied in several medical fields, including as scaffolds for tissue engineering, tissue adhesion, drug delivery systems, coating membranes and various bioabsorbable implants [18–21]. However, fibrin gel alone lacks sufficient mechanical strength for application in bone repair [22]. Therefore, exploiting the adhesive properties of fibrin gel, we herein combined fibrin with the BMP-2/β-TCP complex and investigated its mechanical properties and bone formation ability using a vertical bone reconstruction model in mouse calvaria.

## Materials and methods

### Design and preparation of scaffolds

This study evaluated the following six groups: β-TCP loaded with 6 μg E-rhBMP-2 (low-dose TCP-BMP group), β-TCP loaded with 60 μg E-rhBMP-2 (high-dose TCP-BMP group), fibrin gel loaded with 3 μg E-rhBMP-2 and β-TCP loaded with 3 μg E-rhBMP-2 (low-dose Fibrin-BMP/TCP-BMP group), fibrin gel loaded with 30 μg E-rhBMP-2 and β-TCP loaded with 30 μg E-rhBMP-2 (high-dose Fibrin-BMP/TCP-BMP group), fibrin gel with distilled water (DW) containing 0.5 mM HCl and β-TCP loaded with 6 μg E-rhBMP-2 (low-dose Fibrin-DW/TCP-BMP group), fibrin gel with DW containing 0.5 mM HCl and β-TCP loaded with 60 μg E-rhBMP-2 (high-dose Fibrin-DW/TCP-BMP group).

In the low- and high-dose TCP-BMP groups, E-rhBMP2/β-TCP complex was prepared by mixing 30 mg of porous β-TCP (Superpore, particle size 0.6–1.0 mm, porosity 75(±3)%, HOYA, Tokyo, Japan) with 30 μl of 0.5 mM HCl containing 6 or 60 μg E-rhBMP-2 (Osteopharma Inc., Osaka, Japan). The mixture was incubated for 5 min at room temperature (RT).

The fibrin gel kit, Beriplast^®^ P (CSL Behring K.K, Japan), consisted of two components: Combi-Set A: Human fibrinogen 80 mg/ml, coagulation factor XIII (human) 60 UI/ml, aprotinin (bovine), Combi-Set B: Human thrombin 300 UI/ml, CaCl_2_. Therefore, in the low- and high-dose Fibrin-BMP/TCP-BMP group, to achieve a total of 6 or 60 μg of E-rhBMP-2 in the materials, 24 μl of Combi-Set A solution pre-mixed with 30 μl of 0.5 mM HCl containing 3 or 30 μg E-rhBMP-2 was combined with the E-rhBMP-2/β-TCP complex containing 3 or 30 μg of E-rhBMP-2. The resulting mixture was then combined with Combi-Set B following the same procedure as described above.

In the low- and high-dose Fibrin-DW/TCP-BMP groups, 24 μl of Combi-Set A solution pre-mixed with 30 μl of 0.5 mM HCl was combined with the E-rhBMP-2/β-TCP complex containing 6 or 60 μg of E-rhBMP-2, prepared according to the method described above. Subsequently, 24 μl of Combi-Set B was added, and the mixture was thoroughly stirred before being incubated for 1 min at RT.

Scaffolds of 5 mm in height (*n* = 5 per group) were prepared for mechanical tests.

### Scaffold characterization

Microstructures of TCP-BMP*, Fibrin-BMP*/TCP-BMP*, and scaffolds were freeze-dried and coated with 2% Osmium before observation using a scanning electron microscope (SEM; S-4800, Hitachi High-Tech Corp., Tokyo, Japan). Elemental analysis was performed by energy dispersive X-ray spectroscopy (EDS) using the abovementioned SEM operated at 15 kV. Fourier transform infrared (FT-IR) spectra were obtained using an FT-IR spectrometer (Shimadzu Corp., Kyoto, Japan) with a resolution of 4 cm^−1^.

### Mechanical properties

The mechanical properties of scaffolds were evaluated using a Universal Mechanical Tester (Ez-test; Shimadzu Corp., Kyoto, Japan). Compression tests of the freshly prepared scaffolds were conducted at RT. To ensure accurate results and prevent errors caused by uneven contact surfaces during the loading process, the contact surface of each specimen was gently flattened before testing. For recording, displacement control mode was used with a crosshead displacement rate of 2 mm/min until failure occurred. The compressive stress–strain curves were plotted, and the ultimate stress was determined for each sample using a material testing software (TRAPEZIUM X; Shimadzu Corp.).

### Animal model

C57BL/6J male mice, aged 8–10 weeks, were purchased from CLEA Japan Inc. (Tokyo, Japan). The animal experiment protocol used in this study (OKU-2019254) was approved by the Okayama University Research Committee. Throughout the experimental period, animal care and experimental protocols were performed under the guidelines of the Okayama University Animal Research Committee.

The experimental design was based on a modified vertical bone regeneration model [23]. Under general and local anesthesia, a crescent-shaped cutaneous flap (2 cm) was raised laterally over the mouse calvarium, and the subcutaneous connective tissue was mobilized carefully. The periosteum was dissected from the midline to expose the surgical field of the calvaria surface. The complex scaffolds were positioned into the central region of the calvaria. The incision was sutured in layers using 4-0 nylon monofilament sutures. The mice were then individually housed in cages. Tissues were harvested 4 and 12 weeks after transplantation and fixed with 4% paraformaldehyde solution (PFA; Merck, Kenilworth, NJ, USA) before analysis.

### micro-CT analysis

The fixed calvarial samples were analyzed using a micro-computed tomography (micro-CT) system (SkyScan 1174, Bruker, Kontich, Belgium). The scanning parameters were set to an energy source of 50 kV and 600 μA using a 1-mm filter and a spatial resolution of 17 μm. The region of interest (ROI) was set as the bone regeneration area (hard tissue volume; HV). ROIs were segmented with a global threshold. Bone regeneration height was quantified in mm using the coronal sections using 2D slice analysis software (Data Viewer, Bruker). The bone and β-TCP were analyzed together as ‘hard tissue volume’ using a 3D slice analysis software (CTAn, Bruker).

### Histological analysis

Fixed calvarial samples were decalcified using 10% ethylenediaminetetraacetic acid (EDTA) for 8 days and then embedded in paraffin. Sections (5 µm) were prepared and stained with hematoxylin and eosin (HE) or tartrate-resistant acid phosphatase (TRAP) to analyze the number of osteoclasts. Immunofluorescence (IF) staining for Osterix was performed to evaluate the number of osteoblasts. The IF method was modified according to previous studies and the manufacturer’s instructions [16]. For antigen retrieval, the sections were covered with the L.A.B Solution (Polysciences, Warrington, PA, USA) and incubated for 5 min at RT. The sections were blocked with 5% goat serum for 1 h at 37°C in a humidified chamber. Then, they were incubated with anti-Sp7/Osterix antibody (1:200, ab22552, Abcam, Cambridge, UK) diluted with Signal Booster Immunostain Solution M (BCL-ISM, Beacle, Inc. Kyoto, Japan) at 4°C overnight. Then, sections were incubated with the secondary antibody, Alexa Fluor 647 goat anti-rabbit IgG (1:200, Life Technologies, Gaithersburg, MD, USA) diluted with BCL-ISM for 1 h at RT in a dark chamber. The sections were analyzed under a BZ-X710 fluorescence microscope (Keyence, Osaka, Japan).

The bone volume occupancy rate, residual β-TCP volume occupancy rate, and fibrous tissue volume occupancy rate were determined by calculating the percentage of the corresponding tissue's area relative to the entire regenerated bone area, using ImageJ software (National Institutes of Health, USA). To count the number of osteoblasts and osteoclasts, we segmented the vertical growth into three distinct zones. Within each zone, we randomly captured one image at 20× magnification to serve as the ROI, ensuring to avoid areas proximal to the base (where the new bone has fused with native cranial bone and cannot be distinguished) and the periphery. For each image, three layers were captured: brightfield, DAPI, and Osterix. The brightfield layer delineates the outline of the new bone and was recorded as bone surface. DAPI staining highlights cell nuclei, while Osterix staining identifies osteoblasts. By overlaying the three layers, cells that were double-stained with Osterix and DAPI and located on the bone surface were classified as Osterix-positive osteoblasts. For osteoclasts, multinucleated (≥3) TRAP-positive cells on the bone surface were counted under brightfield microscopy. We conducted quantitative analyses using QuPath 0.4.2, an open-source software [24], to determine the number of osterix-positive osteoblasts per bone surface (N.Ob/BS) and the number of TRAP-positive osteoclasts with more than three nuclei per bone surface (N.Oc/BS).

### Statistical analysis

All the data are shown as mean and standard deviation (SD). Data were statistically processed using Student’s *t*-test, or 1-way ANOVA with a Tukey *post hoc* test to assess significant differences amongst various groups. *P* < 0.05 was considered significant. The software used for data analysis was Prism version 9 (GraphPad Software, Inc., San Diego, CA, USA).

## Result

### Mechanical properties

**Figure 1.**
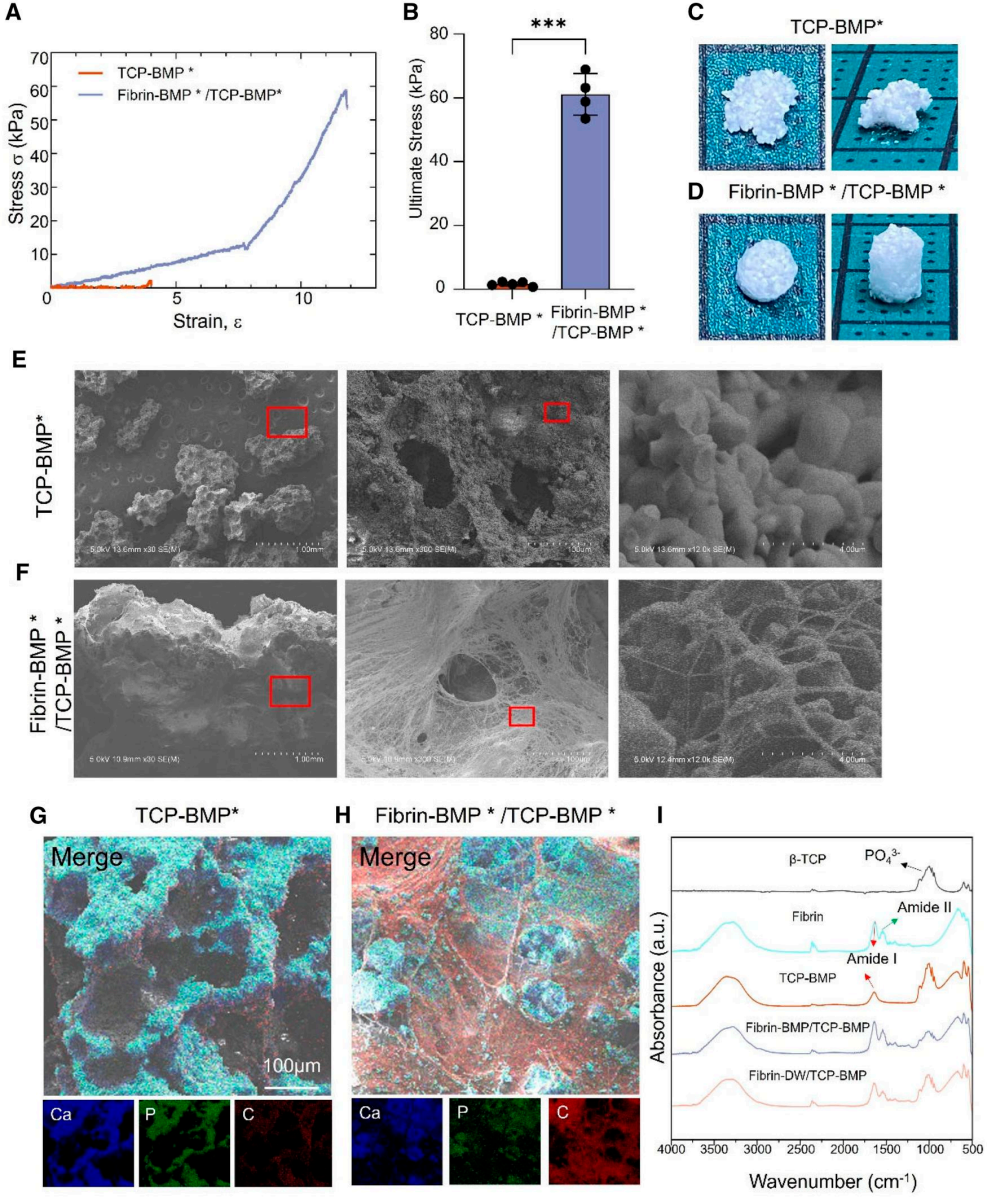
Characterization and mechanical behavior of artificial bone scaffold. (**A**) Representative stress-strain curves of each group, under vertical compression. (**B**) The ultimate stress of TCP-BMP* group (*n* = 5), and Fibrin-BMP*/TCP-BMP* group (*n* = 4). BMP* refers to using water instead of BMP-2, error bars indicate SD, *** *P* < 0.001. (**C**, **D**) Representative images of the specimens from each group for the biomechanical test. (**E**, **F**) SEM image of each group at low (30×) and high magnifications (300×, 12 000×). (**G**, **H**) Energy-dispersive X-ray spectroscopy (EDS) analysis for each group at 200× magnification. The β-TCP is rich in calcium (blue) and phosphorus (green), while fibrin is rich in carbon (red). These three elements were used to distinguish β-TCP and fibrin. (**I**) The FTIR spectra of the β-TCP, Fibrin, TCP-BMP, Fibrin-BMP/TCP-BMP and Fibrin-DW/TCP-BMP groups. Fibrin-BMP/TCP-BMP and Fibrin-DW/TCP-BMP show peaks corresponding to ${\text{PO}}_{4}^{3-}$, amide I and II, which have been identified by the arrows.

The scaffold’s ability to maintain its structure is crucial not only during the initial stages of implantation but also throughout the bone replacement process. Based on general observations, the Fibrin-BMP*/TCP-BMP* group exhibited a more stable and shape-adjustable performance compared to the TCP-BMP* group (Figure 1C and D. BMP* refers to using water instead of BMP-2). Figure 1A shows the stress–strain curves of TCP-BMP* and Fibrin-BMP*/TCP-BMP* scaffolds. TCP-BMP* showed a low strength and mostly a plastic deformation pattern. Fibrin-BMP*/TCP-BMP* featured an initial elastic deformation. However, a sharp increase in stress, corresponding to the structure undergoing densification, was observed in the Fibrin-BMP*/TCP-BMP*. Following densification, the Fibrin-BMP*/TCP-BMP* complex showed a fracture point around 60 kPa, indicating a high strength (Figure 1A).

The mean ultimate compressive strength was 1.7 kPa, 61 kPa for TCP-BMP*, Fibrin-BMP*/TCP-BMP* groups, respectively (Figure 1B). The Fibrin-BMP*/TCP-BMP* group showed significantly higher modulus and compressive strength than the TCP-BMP* group. These data indicate that incorporation of fibrin gel significantly enhanced the elastic modulus and compressive strength *in vitro*.

### Scaffold characterizations

SEM micrographs of the β-TCP particles showed a porous structure (Figure 1E). The fibrin networks and flattened fibrin layers connected adjacent β-TCP particles of the Fibrin-BMP*/TCP-BMP* scaffold, as shown in Figure 1F. In Figure 1F, the fibril formation for fibrin can be observed on the surface and fractured area. Fibrin fibers were found to adhere firmly onto the surface of β-TCP particles. Furthermore, the pores of β-TCP were covered by fibrin. However, the fibrin did not completely encapsulate the β-TCP, leaving some areas of the β-TCP exposed (Figure 1H).

FTIR analysis (Figure 1I) was conducted to confirm the presence of fibrin onto the β-TCP. FTIR spectra of the β-TCP particles showed the characteristic phosphate absorption peaks [25, 26] at 500, 600 and 900–1200 cm^−1^. The spectra of fibrin showed peaks at 1635 and 1540 cm^−1^, which are consistent with the presence of amide bonds found in proteins [27]. The spectra of TCP-BMP showed peaks at 1635 cm^−1^ that are not observed with β-TCP alone. These results show that a certain amount of protein is adsorbed onto the β-TCP, but it was not fibrin. Fibrin-BMP/TCP-BMP, Fibrin-DW/TCP-BMP retained characteristic peaks observed for TCP-BMP in the presence of new peaks for amide II at 1540 cm^−1^ because of the fibrin carbonyl group. Peaks of TCP-BMP were similar to those of Fibrin-BMP/TCP-BMP, and Fibrin-DW/TCP-BMP because of the presence of the same amide group between fibrin and BMP-2.

### Comparative micro-CT and histological analysis 4 weeks post-implantation

**Figure 2.**
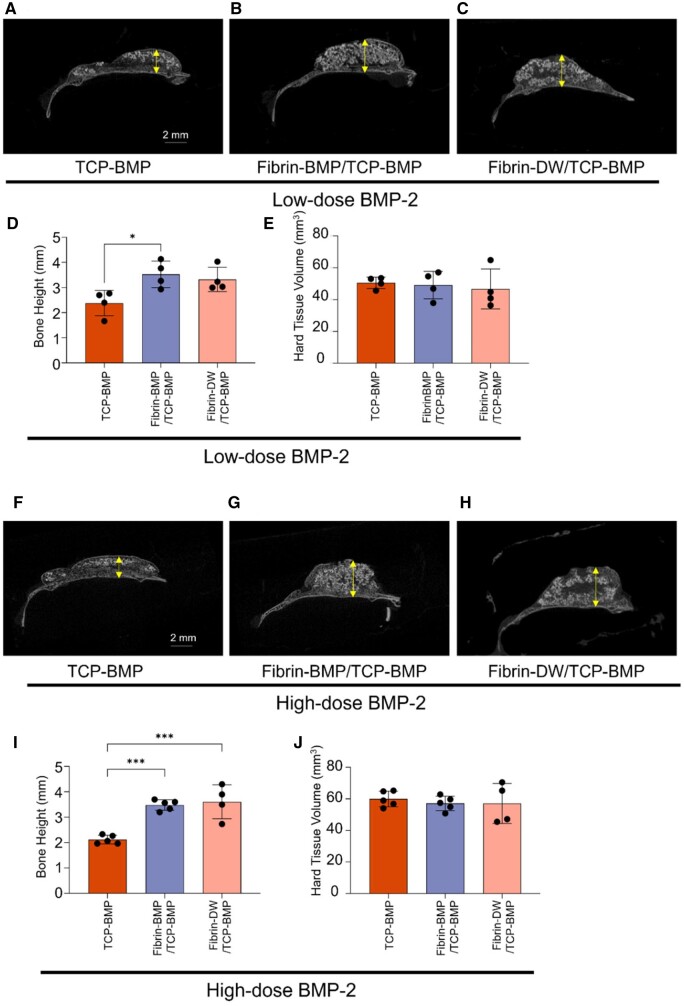
Comparative micro-CT analysis 4 weeks post-implantation. (**A**–**C**) Representative coronal view micro-CT images of a mouse calvaria treated with low-dose (6 μg) BMP-2. A: β-TCP loaded with 6 μg E-rhBMP-2, B: fibrin loaded with 3 μg E-rhBMP-2 and β-TCP loaded with 3 μg E-rhBMP-2, C: fibrin without E-rhBMP-2 and β-TCP loaded with 6 μg E-rhBMP-2. (**D**, **E**) The graph presents the average bone height and hard tissue volume measured from micro-CT analysis data in the low-dose BMP-2 group. Bars represent the means ± SD (*n* = 4). * *P* < 0.05, (1-way ANOVA with post hoc turkey test comparison). (**F**–**H**) Representative coronal view CT images of a mouse calvaria treated with high-dose (60 μg) BMP-2. F: β-TCP loaded with 60 μg E-rhBMP-2, G: fibrin loaded with 30 μg E-rhBMP-2 and β-TCP loaded with 30 μg E-rhBMP-2, H: fibrin without E-rhBMP-2 and β-TCP loaded with 60 μg E-rhBMP-2. (**I**, **J**) The graph presents the average bone height and hard tissue volume measured from micro-CT analysis data in the high-dose BMP-2 group. Bars represent the means ± SD (*n* = 4/5). *** *P* < 0.001 (1-way ANOVA with post hoc turkey test comparison). The arrows in the coronal view micro-CT indicate the site where the height was measured.

In the coronal 2D micro-CT images of the mouse calvaria, the distance from the existing mouse calvaria to the most vertically distant point was measured as the vertical bone formation. At 4 weeks post-implantation, a comparison of the height of bone formation showed no statistically significant difference between the fibrin gel containing BMP-2 (Fibrin-BMP/TCP-BMP group) and the fibrin gel without BMP-2 (Fibrin-DW/TCP-BMP group) in both BMP-2 low-dose and high-dose groups (Figure 2D and I). However, a significant difference was observed when compared to the group without fibrin gel (TCP-BMP group). In other words, these results indicate that fibrin gel is important for the material to retain its shape *in vivo* after 4 weeks of implantation. Of note, regardless of the dosage of BMP-2, there was no statistically significant difference in the volume of hard tissue among the groups (Figure 2E and J). The average hard tissue volume in the high-dose BMP-2 group is roughly 60 mm³, whereas it is only about 50 mm³ in the low-dose BMP-2 group.

**Figure 3.**
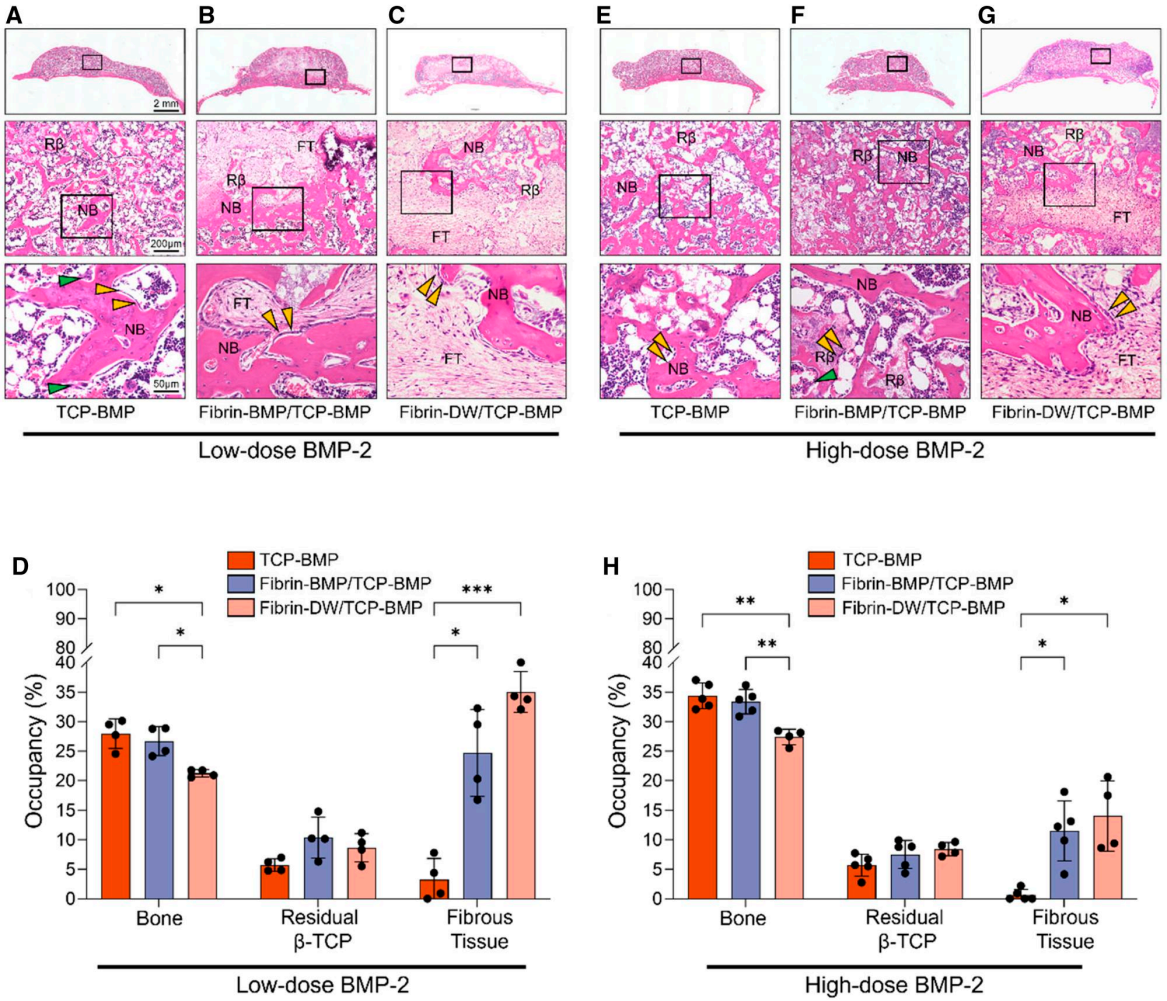
Comparative histological analysis 4 weeks post-implantation. (**A**–**C**) Representative coronal HE staining images of a mouse calvaria treated with low-dose (6 μg) BMP-2. Magnified images for each group are provided below. A: β-TCP loaded with 6 μg E-rhBMP-2. B: fibrin loaded with 3 μg E-rhBMP-2 and β-TCP loaded with 3 μg E-rhBMP-2, C: fibrin without E-rhBMP-2 and β-TCP loaded with 6 μg E-rhBMP-2. (**D**) The graph shows the occupancy of bone, residual β-TCP and fibrous tissue in the regenerated bone for the low-dose (6 μg) BMP-2 group. Bars represent the means ± SD (*n* = 4). * *P* < 0.05, *** *P* < 0.001 (1-way ANOVA with post hoc turkey test comparison). (**E**–**G**) Representative coronal HE staining images of a mouse calvaria treated with high-dose (60 μg) BMP-2. Magnified images for each group are provided below. E: β-TCP loaded with 60 μg E-rhBMP-2. F: fibrin loaded with 30 μg E-rhBMP-2 and β-TCP loaded with 30 μg E-rhBMP-2, G: fibrin without E-rhBMP-2 and β-TCP loaded with 60 μg E-rhBMP-2. (**H**) The graph shows the occupancy of bone, residual β-TCP and fibrous tissue in the regenerated bone for the high-dose (60 μg) BMP-2 group. Bars represent the means ± SD (*n* = 4/5). * *P* < 0.05, ** *P* < 0.01, *** *P* < 0.001 (1-way ANOVA with post hoc turkey test comparison). NB: new bone, Rβ: residual β-TCP, FT: fibrous tissue. Yellow and green arrowheads indicate osteoblast and osteoclast, respectively.

Next, HE staining was performed to evaluate the rate of bone formation and distribution of the newly formed bone. After 4 weeks, abundant new bone formation around the remaining β-TCP and fibrous tissue was observed in all groups (Figure 3A–C and E–G). Interestingly, no significant difference was observed in bone occupancy between the TCP-BMP and Fibrin-BMP/TCP-BMP groups in either low- or high-dose BMP-2. However, bone occupancy in the Fibrin-DW/TCP-BMP group was significantly lower than that in the TCP-BMP and Fibrin-BMP/TCP-BMP groups, especially at high doses of BMP-2 (Figure 3D and H). Furthermore, a continuous bony shell housing well-distributed bone tissue, which was indistinguishable from the host calvaria bone was observed in TCP-BMP and Fibrin-BMP/TCP-BMP groups at both the low and high doses of BMP-2 (Figure 3A, B, E and F). In contrast, in the Fibrin-DW/TCP-BMP group, where the fibrin gel did not contain BMP-2, an intermittent bony shell filled with sparsely distributed bone tissue was observed (Figure 3C and G). Analysis of residual β-TCP and fibrous tissue further revealed that the fibrin gel promotes fibrous tissue formation and does not affect β-TCP resorption, but high doses of BMP-2 can mitigate these effects.

**Figure 4.**
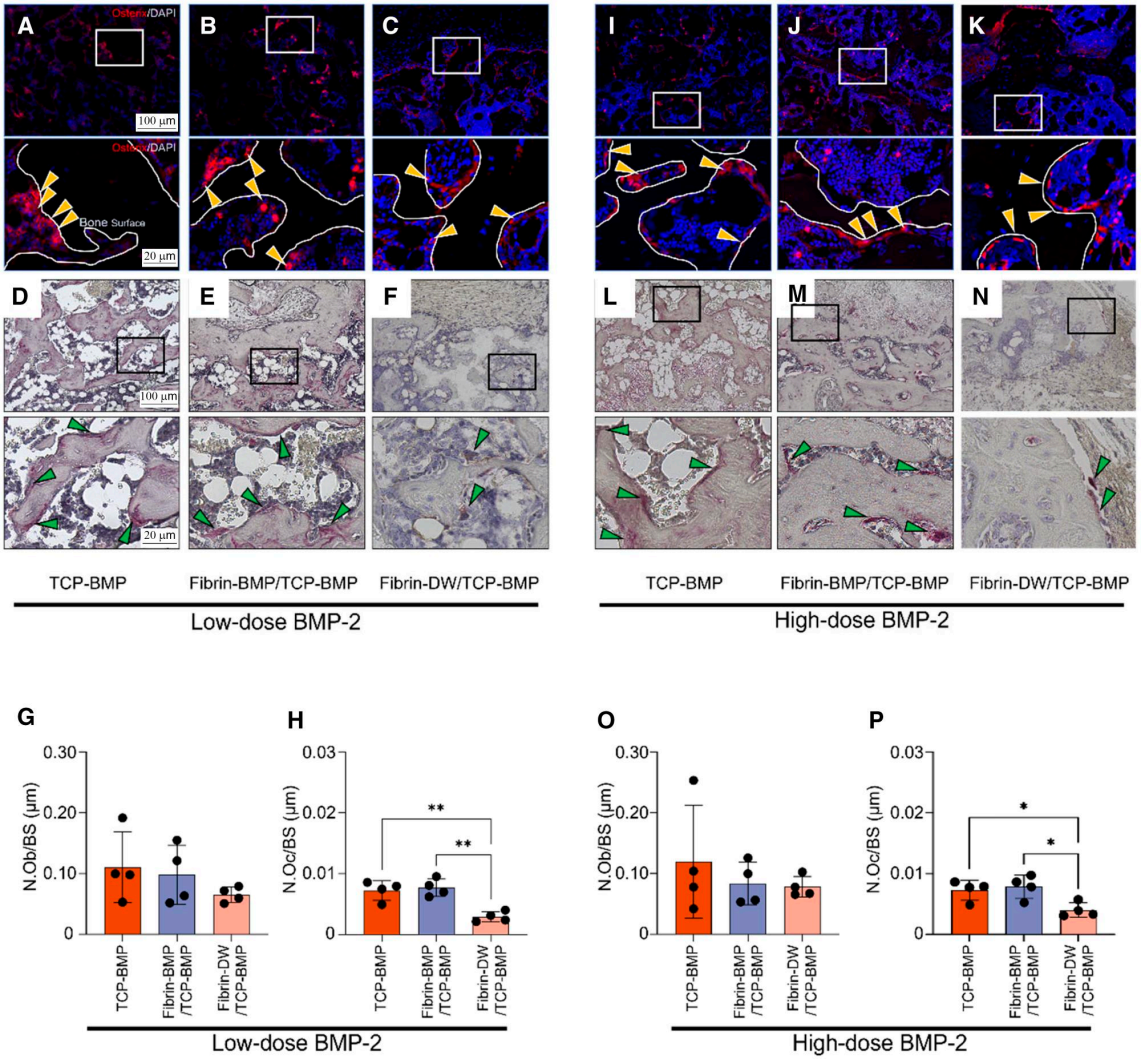
Quantitative analysis of osteoblasts and osteoclasts 4 weeks post-implantation. (**A**–**C**) IHC staining images for Osterix (red) and (**D**–**F**) TRAP staining images of a mouse calvaria treated with low-dose (6 μg) BMP-2. Magnified images for each group are provided below. A, D: β-TCP loaded with 6 μg E-rhBMP-2. B, E: fibrin loaded with 3 μg E-rhBMP-2 and β-TCP loaded with 3 μg E-rhBMP-2. C, F: fibrin without E-rhBMP-2 and β-TCP loaded with 6 μg E-rhBMP-2. (**G**, **H**) The graph shows quantitative analysis of N.Ob/BS (the number of osterix-positive osteoblasts per bone surface in the bone regenerate area) and N.Oc/BS (the number of trap-positive osteoclasts per bone surface in the bone regenerate area). Bars represent the means ± SD (*n* = 4). * *P* < 0.05, ** *P* < 0.01, *** *P* < 0.001 (1-way ANOVA with post hoc turkey test comparison). (**I**–**K**) IHC staining images for Osterix (red) and (**L**–**N**) TRAP staining images of a mouse calvaria treated with high-dose (60 μg) BMP-2. Magnified images for each group are provided below. I, L: β-TCP loaded with 60 μg E-rhBMP-2. J, M: fibrin loaded with 30 μg E-rhBMP-2 and β-TCP loaded with 30 μg E-rhBMP-2, K, N: fibrin without E-rhBMP-2 and β-TCP loaded with 60 μg E-rhBMP-2. (**O**, **P**) The graphs show quantitative analysis of N.Ob/BS and N.Oc/BS. Bars represent the means ± SD (*n* = 4). * *P* < 0.05, ** *P* < 0.01, *** *P* < 0.001 (1-way ANOVA with post hoc turkey test comparison). In the Osterix staining, arrowheads indicate Osterix-positive osteoblasts, and in the TRAP staining, arrowheads indicate TRAP-positive osteoclasts. The curved solid lines indicate the bone surface.

Furthermore, osteoblasts and osteoclasts were evaluated by immunohistochemical (IHC) staining for Osterix (Figure 4A–C and I–K), an osteoblast marker, and TRAP staining (Figure 4D–F and L–N), respectively. As a result, both osteoblasts and osteoclasts were observed to localize around bone and β-TCP. Quantitative analysis revealed that the number of osteoblasts was similar in all groups at both the low and high doses of BMP-2, with no statistically significant difference (Figure 4G and O). On the other hand, significant differences were found in osteoclast numbers; the Fibrin-DW/TCP-BMP group had significantly fewer osteoclasts compared to both the TCP-BMP and Fibrin-BMP/TCP-BMP groups at both the low- and high-doses of BMP-2 (Figure 4H and P).

These results at 4 weeks post-implantation indicated that the addition of fibrin gel effectively helps to preserve the morphology of the scaffold, but simply covering the TCP/BMP-2 surface with fibrin would inhibit bone formation.

### Comparative micro-CT and histological analysis 12 weeks post-implantation

**Figure 5.**
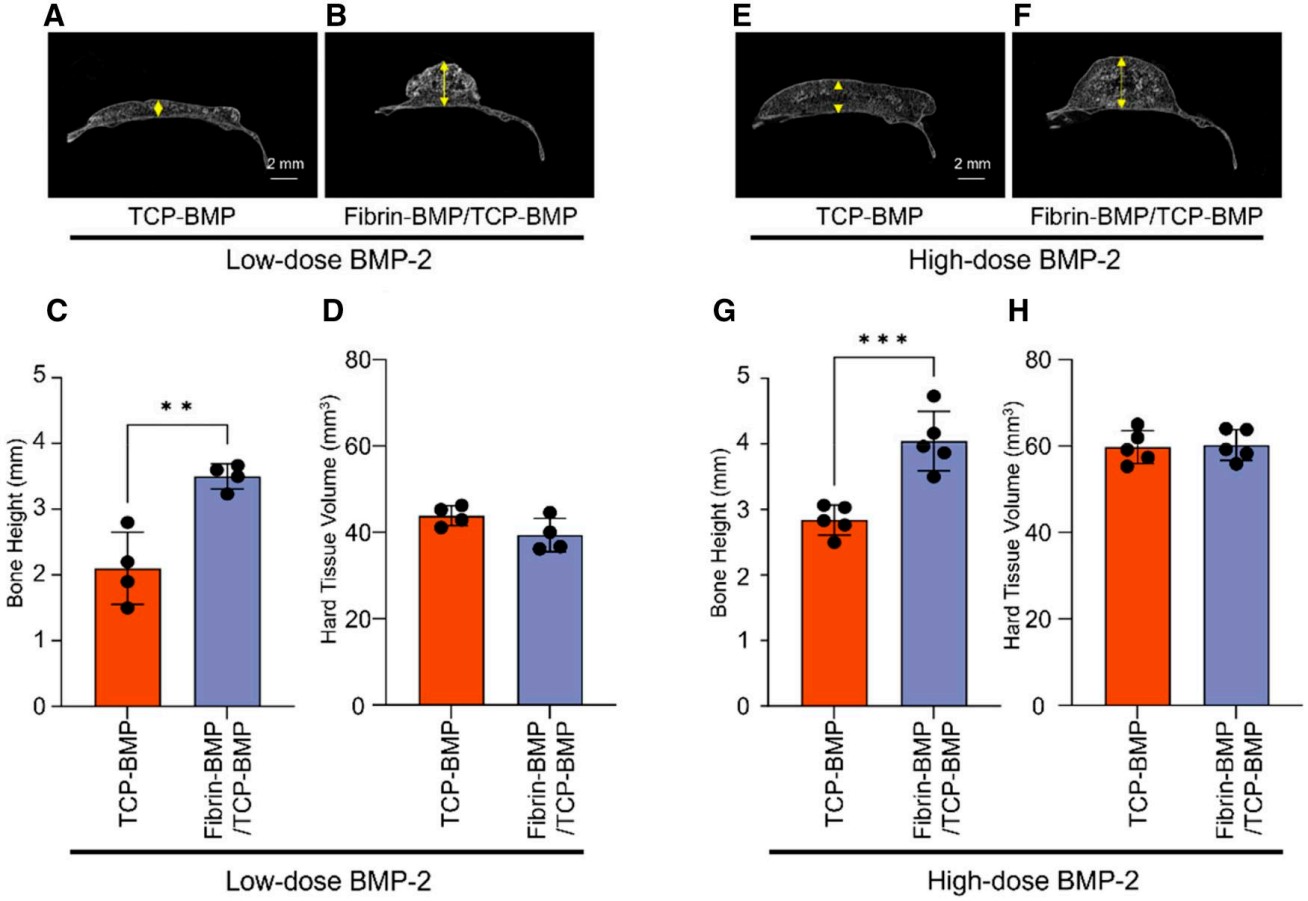
Comparative micro-CT analysis 12 weeks post-implantation. (**A**, **B**) Representative coronal view micro-CT images of a mouse calvaria treated with low-dose (6 μg) BMP-2. A: β-TCP loaded with 6 μg E-rhBMP-2, B: fibrin loaded with 3 μg E-rhBMP-2 and β-TCP loaded with 3 μg E-rhBMP-2. (**E**, **F**) Representative coronal view CT images of a mouse calvaria treated with high-dose (60 μg) BMP-2. E: β-TCP loaded with 60 μg E-rhBMP-2, F: fibrin loaded with 30 μg E-rhBMP-2 and β-TCP loaded with 30 μg E-rhBMP-2. (**C**, **G** and **D**, **H**) The graph presents the average bone height and hard tissue volume measured from micro-CT analysis data in the low and high-dose BMP-2 group. Bars represent the means ± SD (*n* = 4/5). ** *P* < 0.01, *** *P* < 0.001 (Student’s t-test). The arrows in the coronal view micro-CT indicate the site where the height was measured.

Next, since BMP-2 is important to be included in fibrin gel to efficiently induce bone formation, a long-term 12-week observational model was conducted comparing only the TCP-BMP and Fibrin-BMP/TCP-BMP groups while excluding the Fibrin-DW/TCP-BMP group. Micro-CT analysis showed that in all experimental groups, radiopaque images were observed exclusively at the grafted sites, with no ectopic bone formation detected. The height of bone formation was significantly greater in the Fibrin-BMP/TCP-BMP group than in the TCP-BMP group at 12 weeks post-transplantation in both the low- and high BMP-2 doses groups, similar to the results observed at 4 weeks (Figure 5). This indicates that bone morphology remained stable over a long period. With the same dosage of BMP-2, there was no statistically significant difference in the volume of hard tissue between the groups. However, a clear dose-dependent effect was still observed.

**Figure 6.**
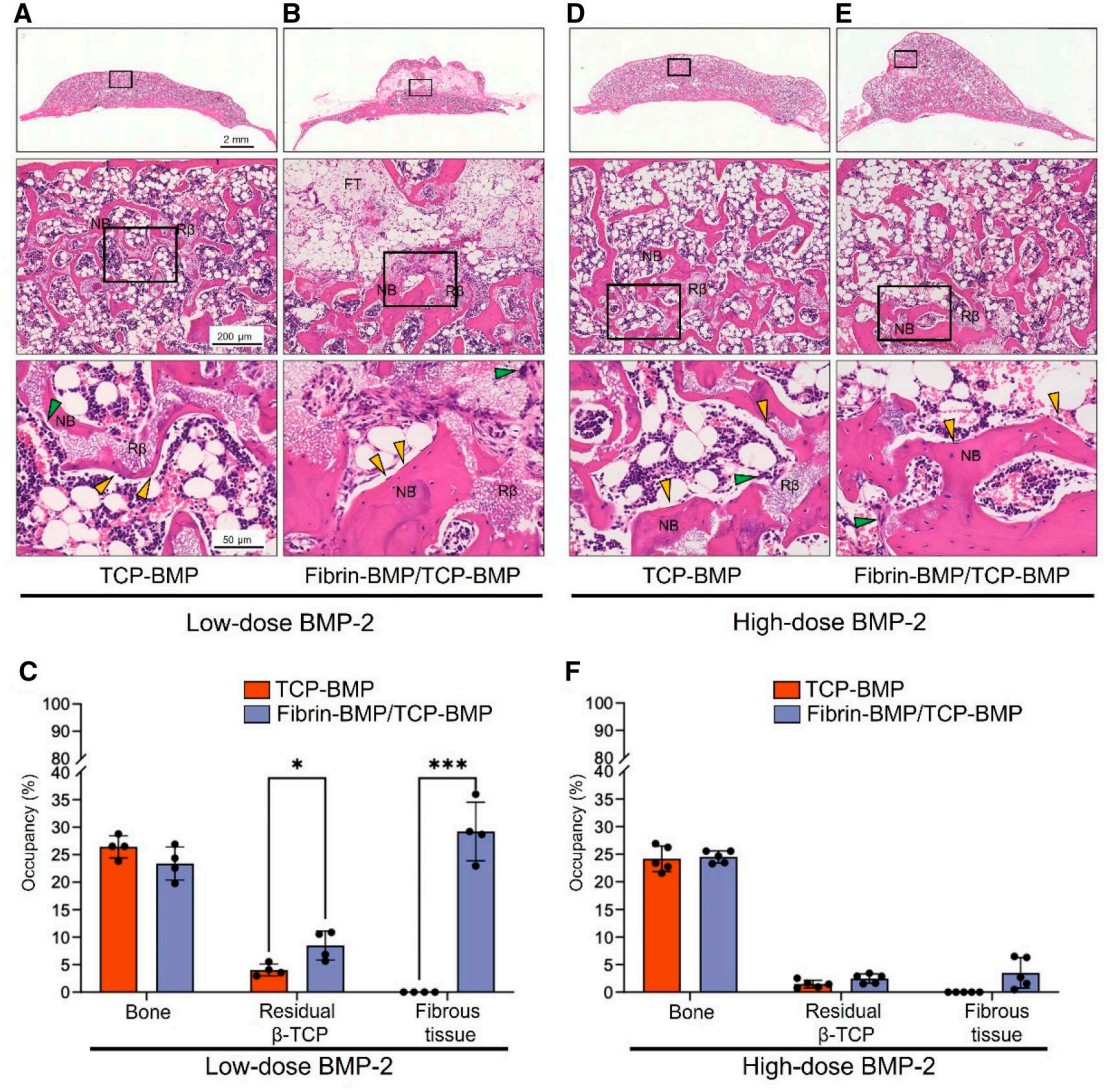
Comparative histological analysis 12 weeks post-implantation. (**A**, **B**) Representative coronal HE staining images of a mouse calvaria treated with low-dose (6 μg) BMP-2. Magnified images for each group are provided below. A: β-TCP loaded with 6 μg E-rhBMP-2. B: fibrin loaded with 3 μg E-rhBMP-2 and β-TCP loaded with 3 μg E-rhBMP-2. (**C**) The graph shows the occupancy of bone, residual β-TCP and fibrous tissue in the regenerated bone for the low-dose (6 μg) BMP-2 group. Bars represent the means ± SD (*n* = 4). * *P* < 0.05, *** *P* < 0.001 (Student’s t-test). (**D**, **E**) Representative coronal HE staining images of a mouse calvaria treated with high-dose (60 μg) BMP-2. Magnified images for each group are provided below. D: β-TCP loaded with 60 μg E-rhBMP-2. E: fibrin loaded with 30 μg E-rhBMP-2 and β-TCP loaded with 30 μg E-rhBMP-2. (**F**) The graph shows the occupancy of bone, residual β-TCP and fibrous tissue in the regenerated bone for the high-dose (60 μg) BMP-2 group. Bars represent the means ± SD (*n* = 5) (Student’s t-test). NB: new bone, Rβ: residual β-TCP, FT: fibrous tissue. Yellow and green arrowheads indicate osteoblast and osteoclast, respectively.

**Figure 7.**
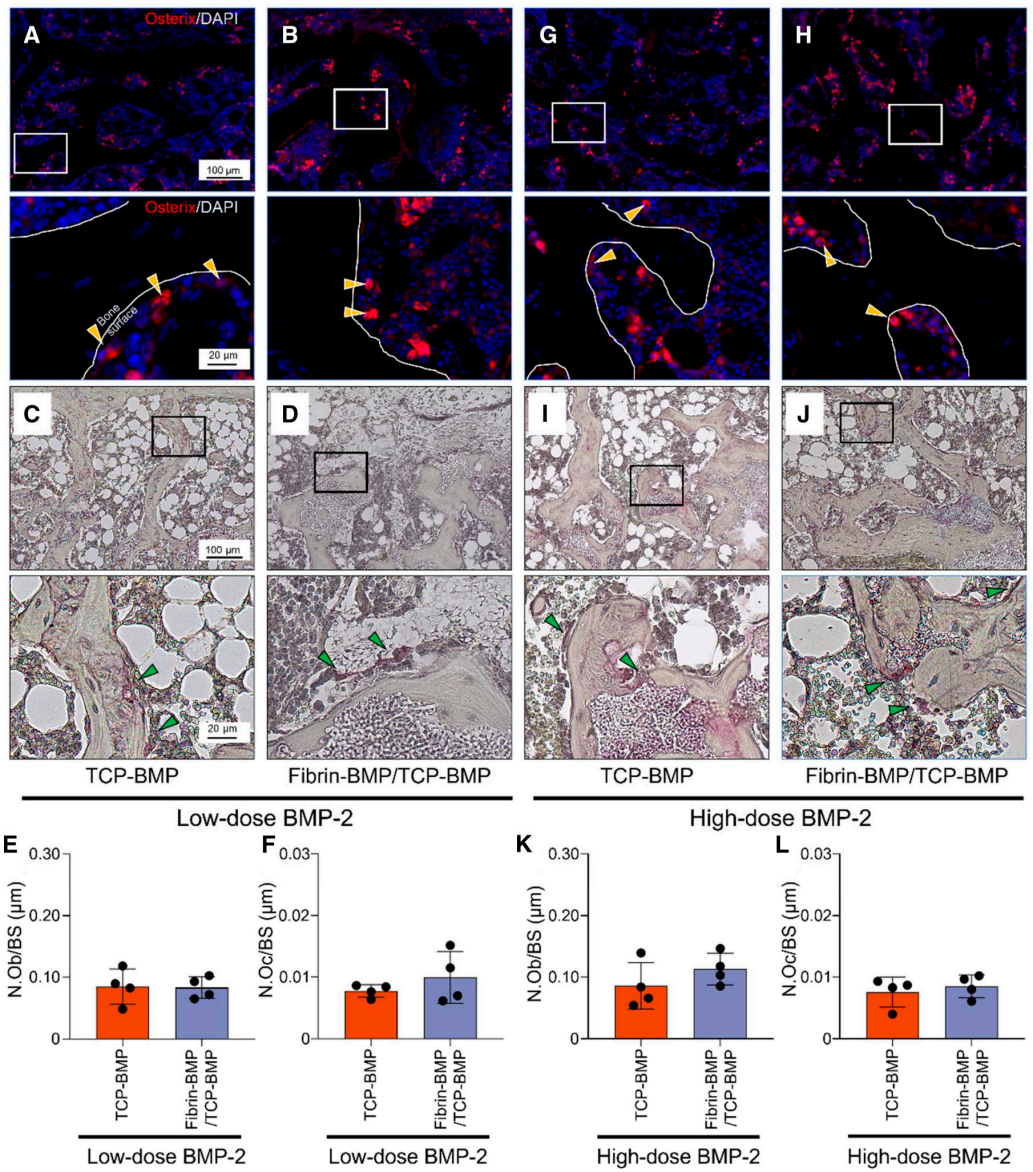
Quantitative analysis of osteoblasts and osteoclasts 12 weeks post-implantation. (**A**, **B**) IHC staining images for Osterix (red) and (**C**, **D**) TRAP staining images of a mouse calvaria treated with low-dose (6 μg) BMP-2. Magnified images for each group are provided below. A, C: β-TCP loaded with 6 μg E-rhBMP-2. B, D: fibrin loaded with 3 μg E-rhBMP-2 and β-TCP loaded with 3 μg E-rhBMP-2. (**E**, **F**) The graph shows quantitative analysis of N.Ob/BS (the number of osterix-positive osteoblasts per bone surface in the bone regenerate area) and N.Oc/BS (the number of trap-positive osteoclasts per bone surface in the bone regenerate area). Bars represent the means ± SD (*n* = 4) (Student’s t-test). (**G**, **H**) IHC staining images for Osterix (red) and (**I**, **J**) TRAP staining images of a mouse calvaria treated with high-dose (60 μg) BMP-2. Magnified images for each group are provided below. G, I: β-TCP loaded with 60 μg E-rhBMP-2. H, J: fibrin loaded with 30 μg E-rhBMP-2 and β-TCP loaded with 30 μg E-rhBMP-2. (**K**, **L**) The graph shows quantitative analysis of N.Ob/BS and N.Oc/BS. Bars represent the means ± SD (*n* = 4) (Student’s t-test). In the Osterix staining, arrowheads indicate Osterix-positive osteoblasts, and in the TRAP staining, arrowheads indicate TRAP-positive osteoclasts. The curved solid lines indicate the bone surface.

Histological analysis revealed no significant difference in bone occupancy between the TCP-BMP and Fibrin-BMP/TCP-BMP groups in both the low and high-dose BMP-2 groups (Figure 6C and F). However, in the low-dose BMP-2 group, a substantial amount of fibrous tissue remained in the Fibrin-BMP/TCP-BMP group, and significantly more β-TCP was retained in the Fibrin-BMP/TCP-BMP group compared to the TCP-BMP group (Figure 6A–C). Interestingly, in the high-dose BMP-2 group, fibrous tissue was almost unobservable in the Fibrin-BMP/TCP-BMP group, and there was no significant difference in the occupancy rates of residual β-TCP and fibrous tissue compared to the TCP-BMP group. Note that the regenerated bone was almost completely filled with bone and bone marrow tissue (Figure 6D–F). There was no significant difference in the number of osteoblasts and osteoclasts between the TCP-BMP and Fibrin-BMP/TCP-BMP groups in both the low- and high-dose BMP-2 groups (Figure 7E–L).

These results indicate that over 12 weeks, the Fibrin-BMP/TCP-BMP group was able to maintain the vertically formed bone morphology. Furthermore, in the Fibrin-BMP/TCP-BMP group with high-dose BMP-2, both β-TCP were almost completely absorbed, indicating that the regenerated bone was almost entirely filled with bone and bone marrow.

## Discussion

For clinical bone repair purposes, collagen sponge is the only carrier in combination with BMP-2 approved to be used [28]. However, the low natural affinity between BMP-2 and collagen sponge can lead to adverse side effects, such as ectopic bone formation [29], osteoclastic resorption [30] or lack of space-maintaining capacity [31]. Composite bone repair scaffolds hold promise for orthopedics and dentistry, offering innovative approaches for clinical treatment strategies. Although various composite scaffolds are being developed, they face production challenges that hinder their clinical translation [32–34]. Our previous research indicated that β-TCP and E-rhBMP-2 scaffolds exhibit equivalent osteogenic ability to the gold standard of autologous bone grafting in the canine alveolar bone defect model [16]. Nevertheless, they are challenging to use for maintaining the desired surgical shape and pose a risk of ectopic bone formation if displaced from the implantation site, thereby requiring careful surgical application. Some studies have used poly(lactic-co-glycolic acid) (PLGA) to enhance the mechanical strength of the scaffold [32, 35]. However, the fabrication process is complex and time-consuming, making it less feasible for widespread clinical application. To address these issues, we herein assessed the effect of fibrin gel on maintaining graft morphology and its role in bone formation when combined with β-TCP and E-rhBMP-2 in a mouse model. The addition of fibrin gel significantly enhanced the compressive strength and maintained bone morphology for up to 12 weeks post-implantation. When used with a high dose of BMP-2, β-TCP was almost completely resorbed and had little fibrous tissue, leading to the formation of a mature bone densely filled with bone and bone marrow. The disadvantages of β-TCP/E-rhBMP-2 scaffolds were supplanted, and bone regeneration was not suppressed by the application of rhBMP-2 to a fibrin widely used in clinical practice.

In dental practice, large vertical resorption of the alveolar bone complicates implant procedures [36, 37]. Traditional methods require the use of nonabsorbable membranes, such as titanium membranes, to maintain the graft morphology, complicating the surgery [38]. However, if the graft materials could be hardened post-implantation to withstand gingival suture pressure, it would simplify the process and benefit both patients and surgeons. Fibrin-based products are biodegradable materials and are extensively used in tissue engineering scaffolds as drug-loaded materials [19]. Fibrin alone is insufficient for osteogenesis [22]. However, when BMP-2 or bone powder is added to fibrin, it exhibits excellent osteogenic potential [22, 39]. Koolen *et al.* reported that when bone defects were treated with fibrin-embedded BMP-2, the defects were filled with bone in a few weeks and the regenerated bone showed active remodeling [22]. However, they also reported that the BMP-2-containing fibrin gels are difficult to maintain their morphology at the graft site and are not effective in bone defects submitted to load-bearing conditions [22]. Since the combination of β-TCP and BMP-2 has already been commercialized and its clinical application has started, we aimed to improve the mechanical strength by combining fibrin gel with BMP-2/β-TCP, while keeping the BMP-2/β-TCP combination unchanged. In fact, the addition of fibrin gel to BMP-2/β-TCP significantly increased its mechanical strength. However, in a mouse calvaria transplantation model, which mimics the vertical bone formation of the jawbone, the ability of the scaffold to maintain its shape was not significantly different between the Fibrin-BMP/TCP-BMP and Fibrin-DW/TCP-BMP groups at 4 weeks, when comparing the height of the newly formed bone (Figure 2). This suggests that the addition of fibrin gel to BMP-2/β-TCP can preserve graft morphology without compromising the surgical handling of the graft. Future studies will be necessary to verify whether the mechanical strength of this material is adequate for vertical bone augmentation of the jawbone in larger animals.

The osteoinductive properties of BMP-2 stimulate osteoblast and osteoclast activities, leading to more robust and accelerated bone matrix formation and remodeling [14, 40]. In turn, this increased bone formation and turnover accelerate the resorption of the β-TCP and fibrin gel, facilitating the replacement of the scaffold material with newly formed physiological bone [41–43]. Moreover, the amount of BMP-2 is crucial in modulating these processes [44–46]. Wei *et al.* [47] conducted experiments on rats with critical-sized calvarial defects, filling them with BMP-2 functionalized β-TCP loaded with gradient doses of BMP-2 (ranging from 0 to 300 μg/g). Their findings indicated that over 150 μg/g was effective for bone formation, which is consistent with our previous experiments on the pig model [13, 47]. Therefore, we used a ratio of 6 μg BMP-2 per 30 mg β-TCP, equivalent to 200 μg/g, which was set as the low dosage group. In our previous study using a pig model, a ratio of 1000 μg/g achieved the highest bone volume density. However, considering that new materials were added to the experimental group and according to earlier investigations in mouse models [48, 49], BMP-2 was coated onto β-TCP in a single dose of 60 μg per 30 mg of β-TCP, which corresponds to a ratio of 2000 μg/g, and was set as the high dosage group. The experimental groups were divided into two subgroups based on whether fibrin contained BMP-2 or not, while keeping the total amount of BMP-2 constant in each group. In this study, higher amounts of BMP-2 enhanced the rate of scaffold resorption and formed higher bone volume, which corresponded with the accelerated formation of physiological bone. This dose-dependent effect is beneficial as it allows for more controlled and efficient integration of the scaffold into the host tissue, ensuring that the regenerated bone is both functionally and structurally similar to natural bone. Furthermore, the concentration-dependent increase in bone formation and decrease in the amount of fibrous tissue in the Fibrin-BMP/TCP-BMP and Fibrin-DW/TCP-BMP groups suggest that the dose of BMP-2 plays an important role in bone formation.

Four weeks after implantation, clear differences in bone formation and cellular activity were observed among TCP-BMP, Fibrin-BMP/TCP-BMP and Fibrin-DW/TCP-BMP groups. Notably, less bone formation and an uneven spatial distribution of new bone were evident in the Fibrin-DW/TCP-BMP groups compared to the other groups, despite there being no significant difference in the number of osteoblasts between the groups (Figure 4G and O). From the SEM results, adding fibrin to β-TCP can affect the pore size of the material by adsorbing fibrin to β-TCP. However, the osteogenic ability of β-TCP is influenced by interconnecting channels, porosity, and, most importantly, pore size, which determines the capacity of bone ingrowth into the porous scaffold [50]. This may be because the presence of fibrin on the β-TCP surface delayed the release of BMP-2. Additionally, fibroblasts were grown in fibrin [51], which in turn inhibited bone formation [52]. Another possible explanation is that while BMP-2 may not directly affect osteoblast proliferation in the short term, its presence likely enhances the activity of osteoblasts, thereby inducing more effective bone formation without affecting its number. However, a deeper understanding of the functional activity of osteoblasts and the status of new bone formation would require the use of methods such as calcein double labeling to evaluate osteoblast activity [53, 54], which remains a task for future research. On the other hand, the number of osteoclasts was significantly reduced in the Fibrin-DW/TCP-BMP groups. It is well known that BMP-2 not only acts directly on osteoblasts but also on osteoclasts, regulating bone remodeling [14, 40]. The reduction in osteoclast numbers could be due to insufficient osteoinductive signaling in the absence of BMP-2, leading to decreased bone resorption activity and slower turnover of bone matrix [55–57] (Figure 4H and P). Future research should increase the frequency of observations, which may reveal the growth curve of osteoclasts. In contrast, the Fibrin-BMP/TCP-BMP groups, where fibrin was rich in BMP-2, allowed effective bone transformation. Moreover, the Fibrin-BMP/TCP-BMP showed no significant difference in the number of osteoblasts or osteoclasts at both 4- and 12-weeks post-transplantation when compared with the positive control group of TCP-BMP (Figures 4 and 7). This indicates that the inclusion of BMP-2 in the fibrin gel may eliminate the suppression of bone remodeling that occurs when fibrin is used alone, resulting in bone remodeling similar to that observed in the control group.

This study is the first to use E-rhBMP-2-functionalized β-TCP particles and fibrin to investigate vertical bone formation in a mouse calvaria model. The application of E-rhBMP-2 to fibrin, along with the incorporation of β-TCP/E-rhBMP-2, allows clinicians to easily manipulate these materials and maintain them in a preferred space and shape for clinical applications.

However, this study still has several limitations. First, the release kinetics of rhBMP-2 from Fibrin-BMP/TCP-BMP and Fibrin-DW/TCP-BMP were not investigated. Previous studies [22, 58, 59] have reported on the release kinetics of fibrin and β-TCP, showing that approximately 50% of BMP-2 remains in β-TCP on the first day, followed by a gradual release over more than 14 days in both *in vivo* and *in vitro* settings [58, 59]. The release kinetics of the fibrin/BMP-2 complex exhibited variability, with some profiles closely mirroring the release from β-TCP [59], while others maintaining a consistent slow release over 28 days [22, 60], potentially influenced by added additives. Overall, both carriers exhibit superior sustained release compared to the absorbable collagen sponge currently used in clinical trials [61]. Second, the results obtained from rodent models cannot be directly extrapolated to humans due to the species-specific concentration requirements of E-rhBMP-2 for osteogenesis [46]. Additionally, the dosage of E-rhBMP-2 used in this study was higher than that in most other studies [62]. Even if the highest dose of 60 μg of E-rhBMP-2 were to enter the bloodstream all at once, the total systemic exposure would still be well below the thresholds observed in preclinical drug toxicity studies [63]. However, due to its controlled release pattern, the ErhBMP-2 primarily exerted its effects locally, with minimal entry into the systemic circulation, and no adverse effects such as skin erythema, ulceration or mortality. Nevertheless, future clinical trials and preclinical studies with larger animal models are necessary to further investigate the safety and efficacy of E-rhBMP-2-functionalized β-TCP and fibrin.

## Conclusion

We initially attempted to achieve vertical bone regeneration using a β-TCP/BMP-2 composite optimized with plain fibrin gel. The results demonstrated that the fibrin gel significantly improves the mechanical strength and surgical manageability but suppresses the bone formation ability of E-rhBMP-2-adsorbed β-TCP scaffold. However, by optimizing the distribution of BMP-2 while maintaining the total BMP-2 amount constant and using Fibrin-BMP/TCP-BMP composite, we successfully achieved vertical bone regeneration. For clinical applications, it may be advisable to directly apply the Fibrin-rhBMP-2/TCP-BMP composite within or onto the bone defect. This approach enables clinicians to easily achieve and preserve the desired spatial configuration and morphology of the graft.
